# Algorithmic differentiation improves the computational efficiency of OpenSim-based trajectory optimization of human movement

**DOI:** 10.1371/journal.pone.0217730

**Published:** 2019-10-17

**Authors:** Antoine Falisse, Gil Serrancolí, Christopher L. Dembia, Joris Gillis, Friedl De Groote

**Affiliations:** 1 Department of Movement Sciences, KU Leuven, Leuven, Belgium; 2 Department of Mechanical Engineering, Universitat Politècnica de Catalunya, Barcelona, Catalunya, Spain; 3 Department of Mechanical Engineering, Stanford University, Stanford, California, United States of America; 4 Department of Mechanical Engineering, KU Leuven, Leuven, Belgium; 5 DMMS Lab, Flanders Make, Leuven, Belgium; The Ohio State University, UNITED STATES

## Abstract

Algorithmic differentiation (AD) is an alternative to finite differences (FD) for evaluating function derivatives. The primary aim of this study was to demonstrate the computational benefits of using AD instead of FD in OpenSim-based trajectory optimization of human movement. The secondary aim was to evaluate computational choices including different AD tools, different linear solvers, and the use of first- or second-order derivatives. First, we enabled the use of AD in OpenSim through a custom source code transformation tool and through the operator overloading tool ADOL-C. Second, we developed an interface between OpenSim and CasADi to solve trajectory optimization problems. Third, we evaluated computational choices through simulations of perturbed balance, two-dimensional predictive simulations of walking, and three-dimensional tracking simulations of walking. We performed all simulations using direct collocation and implicit differential equations. Using AD through our custom tool was between 1.8 ± 0.1 and 17.8 ± 4.9 times faster than using FD, and between 3.6 ± 0.3 and 12.3 ± 1.3 times faster than using AD through ADOL-C. The linear solver efficiency was problem-dependent and no solver was consistently more efficient. Using second-order derivatives was more efficient for balance simulations but less efficient for walking simulations. The walking simulations were physiologically realistic. These results highlight how the use of AD drastically decreases computational time of trajectory optimization problems as compared to more common FD. Overall, combining AD with direct collocation and implicit differential equations decreases the computational burden of trajectory optimization of human movement, which will facilitate their use for biomechanical applications requiring the use of detailed models of the musculoskeletal system.

## Introduction

Combining musculoskeletal modeling and dynamic simulation is a powerful approach to study the mechanisms underlying human movement. In the last decades, researchers have primarily used inverse dynamic simulations to identify biomechanical variables (e.g., muscle forces and joint loads) underlying observed movements. Yet dynamic simulations can also be applied to generate novel movements. Such predictive simulations have the potential to reveal cause-effect relationships that cannot be explored based on inverse dynamic simulations that require movement kinematics as input. Novel movements can be generated by solving trajectory optimization problems. Generally, trajectory optimization consists of identifying a trajectory that optimizes an objective function subject to a set of dynamic and path constraints [1]. In the biomechanical field, researchers have used trajectory optimization for solving two main types of problems. In tracking problems, the objective function is the difference between a variable’s measured and simulated value [2–4], whereas in predictive problems, the objective function represents a movement related performance criterion (e.g., minimizing muscle fatigue) [5–8]. However, the nonlinearity and stiffness of the dynamic equations characterizing the musculoskeletal system cause the underlying optimal control problems to be challenging to solve and computationally expensive [5,7,8]. For example, small changes in controls can cause large changes in kinematics and hence a foot to penetrate into the ground, drastically increasing ground reaction forces. These challenges have caused the biomechanics community to primarily perform studies based on inverse dynamic analyses of observed movements rather than trajectory optimization of novel movements.

Over the last decade, the increase in computer performance and the use of efficient numerical methods have equipped researchers with more efficient tools for solving trajectory optimization problems. In particular, direct collocation methods [4,6,8–11] and implicit formulations of the musculoskeletal dynamics [10,12] have become popular. Direct collocation reduces the sensitivity of the objective function to the optimization variables, compared to other methods such as direct shooting [5], by reducing the time horizon of the integration. Direct collocation converts optimal control problems into large sparse nonlinear programming problems (NLPs) that readily available NLP solvers (e.g., IPOPT [13]) can solve efficiently. Implicit formulations of the musculoskeletal dynamics improve the numerical conditioning of the NLP over explicit formulations by, for example, removing the need to divide by small muscle activations [10] or invert a mass matrix that is near-singular due to body segments with a large range of masses and moments of inertia [12]. In implicit formulations, additional controls are typically introduced for the time derivative of the states, which allows imposing the nonlinear dynamic equations as algebraic constraints in their implicit rather than explicit form (i.e., $\dot{y}=u,$ 0 = *f*_*i*_(*y*,*u*) instead of $\dot{y}=f_{e}(y)$).

**Fig 1:**
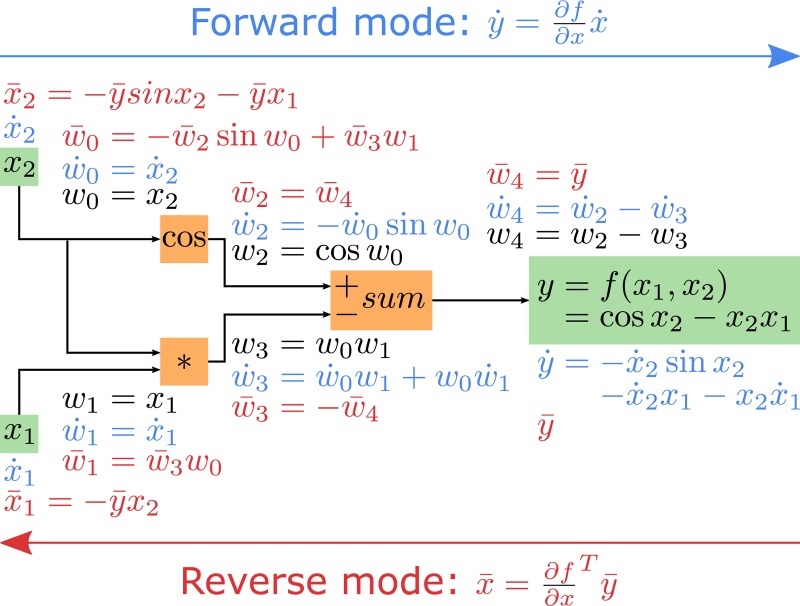
Example of AD forward and reverse modes. A function *y* = *f*(*x*_1_,*x*_2_) = *cos x*_2_−*x*_2_*x*_1_ is broken down into a sequence of elementary operations, forming an expression graph. In the forward mode, the forward seeds ${\dot{x}}_{1}$ and ${\dot{x}}_{2}$ are propagated from the inputs to the output, and the Jacobian *J* = ∂*f*/∂***x*** relates ${\dot{x}}_{1}$ and ${\dot{x}}_{2}$ to the forward sensitivity $\dot{y}$. In the reverse mode, the reverse seed $\bar{y}$ is propagated from the output to the inputs, and the transposed Jacobian *J*^*T*^ relates $\bar{y}$ to the reverse sensitivities ${\bar{x}}_{1}$ and ${\bar{x}}_{2}$.

Algorithmic differentiation (AD) is another numerical tool that can improve the efficiency of trajectory optimization [14,15]. AD is a technique for evaluating derivatives of functions represented by computer programs. It is, therefore, an alternative to finite differences (FD) for evaluating the derivative matrices required by the NLP solver, namely the objective function gradient, the constraint Jacobian, and the Hessian of the Lagrangian (henceforth referred to as simply Hessian). These evaluations are obtained free of truncation errors, in contrast with FD, and for a computational cost of the same order of magnitude as the cost of evaluating the original function. AD relies on the observation that any function can be broken down into a sequence of elementary operations, forming an expression graph (example in Fig 1). AD then relies on the chain rule of calculus that describes how to calculate the derivative of a composition of functions [15]. By traversing a function’s expression graph while applying the chain rule, AD allows computing the function derivatives. Note that, like FD, AD can exploit the sparsity of the aforementioned derivative matrices resulting, for example, from applying direct collocation [16].

AD allows traversing the expression graph in two directions or modes: from the inputs to the outputs in its forward mode and from the outputs to the inputs in its reverse mode. This permits the evaluation of two types of directional derivatives: Jacobian-times-vector product and Jacobian-transposed-times-vector product in the forward and reverse mode, respectively. The computational efficiency of the AD mode depends on the problem dimensions. Consider the function $G:R^{n}\to R^{m}:y=G(x)$ describing the *m* NLP constraints *y* as a function of the *n* optimization variables *x*. The constraint Jacobian *J* = ∂*y*/∂*x* is a matrix with size *m x n*. In the forward mode, *J* relates forward seeds $\dot{x}$ to forward sensitivities $\dot{y}:\;\dot{y}=J\dot{x}$ (example in Fig 1). In the reverse mode, *J*^*T*^ relates reverse seeds $\bar{y}$ to reverse sensitivities $\bar{x}:\;\bar{x}=J^{T}\bar{y}$ (example in Fig 1). In the forward mode, the cost of evaluating *J* is proportional to *n* times the cost of evaluating *G*. In the reverse mode, the cost of evaluating *J*^*T*^ is proportional to *m* times the cost of evaluating *G*. If there are many more inputs *n* than outputs *m*, the reverse mode may drastically decrease the number of function evaluations required to evaluate *J* and highly reduce the computational time (CPU time) as compared to the forward mode [15,17].

Two main approaches exist for adding AD to existing software, namely operator overloading and source code transformation. Source code transformation is inherently faster than operator overloading but may not be readily available for all features of a programming language. In the operator overloading approach, AD’s algorithms are applied after the evaluation of the original function using concrete numerical inputs. This is typically performed by introducing a new numerical type that stores information about partial derivatives as calculations proceed (e.g., through operator overloading in C++) [15,17]. Examples of AD tools using operator overloading in C++ are ADOL-C [18] and CppAD [19]. In the source code transformation approach, the AD tool analyzes a given function’s source code and outputs a new function that computes the forward or reverse mode of that function. Examples of AD tools using source code transformation are ADiGator for MATLAB [20] and CasADi that is available for C++, Python, and MATLAB [21]. CasADi is a modern actively developed tool for nonlinear optimization and AD that has many additional features (e.g., code generation) and interfaces with NLP solvers designed to handle large and sparse NLPs (e.g., IPOPT). CasADi provides a high-level, symbolic, way to construct an expression graph, on which source code transformation is applied. The resultant expression graph can be code-generated to achieve the computational efficiency of pure source code transformation.

AD has a long history [14] but has rarely been applied in biomechanics, likely because AD is relatively unknown in the field and is not integrated as part of widely used biomechanical software packages. In previous work, we solved muscle redundancy problems while exploiting AD [10,22]. For this purpose, we used GPOPS-II [23], a MATLAB software for solving optimal control problems with direct collocation, in combination with ADiGator. However, these problems were limited to models implemented in MATLAB, enabling the use of ADiGator. Generating simulations of human movement requires expanding these problems to account for the multi-body dynamics. OpenSim [24,25] and its dynamics engine Simbody [26] are widely used open-source software packages for musculoskeletal modeling and biomechanical dynamic simulation. These packages provide multi-body dynamics models and have been used for trajectory optimization of human gait [3,4,8,11]. Yet they currently do not leverage tools for AD. Moreover, they are written in C++, which would prevent the use of ADiGator.

AD is increasingly used for trajectory optimization in related fields such as rigid body dynamics for robotic applications and several software packages leverage AD tools [27]. RobCoGen is a modeling tool for rigid body dynamics that supports AD through source code transformation. Giftthaler *et al*. showed that trajectory optimization of gait for a quadrupedal robot modeled with RobCoGen was five times faster with AD than with FD [27]. Other packages for robotic applications with modules supporting AD include Drake [28], Robotran [29], MBSlib [30], and Pinocchio [31]. Drake is a collection of tools that relies on Eigen [32] for linear algebra. Eigen has a module supporting AD’s forward mode using operator overloading. Robotran is a symbolic software to model multibody systems that can be interfaced with CasADi to solve optimal control problems. MBSlib is a multibody system library supporting AD through ADOL-C. Finally, Pinocchio is a software platform implementing algorithms for rigid body dynamics that can be interfaced with ADOL-C, CppAD, and CasADi. Note that AD is not exclusively used for trajectory optimization and is also applied in other related fields including deep learning with libraries such as TensorFlow [33] and Theano [34], and applications for robotic gait optimization (e.g., [35]).

The contribution of this study is threefold. First, we enabled the use of AD in OpenSim and Simbody (henceforth referred to as OpenSim). We compared two approaches: we incorporated the operator overloading AD tool ADOL-C and we developed our own AD tool Recorder that uses operator overloading to construct an expression graph on which source code transformation is applied using CasADi. Second, we interfaced OpenSim with CasADi, enabling trajectory optimization using OpenSim’s multi-body dynamics models while benefitting from CasADi’s efficient interface with NLP solvers. Third, we evaluated the efficiency of different computational choices based on trajectory optimization problems of varying complexity solved with IPOPT. We compared three different derivative scenarios: AD with ADOL-C, AD with Recorder, and FD. In addition, we compared different linear solvers and different Hessian calculation schemes within IPOPT, to aid users in choosing the most efficient solver settings. Primal-dual interior point methods such as IPOPT rely on linear solvers to solve the primal-dual system, which involves the Hessian, when computing the Newton step direction during the optimization [36]. The Hessian can be exact (i.e., based on second-order derivative information) or approximated with a limited-memory quasi-Newton method (L-BFGS) that only requires first-order derivative information. We found that using AD through Recorder was more efficient than using FD or AD through ADOL-C, whereas the efficiency of the linear solver and Hessian calculation scheme was problem-dependent.

## Materials and methods

### Tools to enable the use of AD in OpenSim

**Fig 2:**
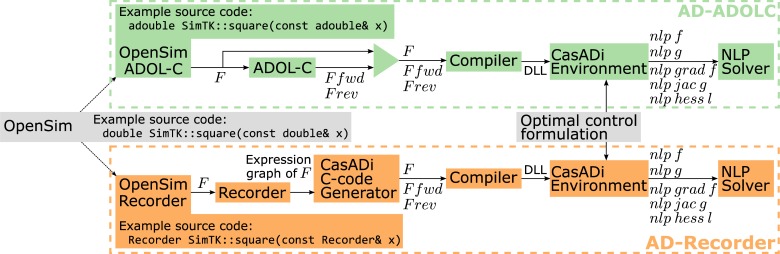
Flowchart depicting the optimal control framework. We developed two approaches (AD-ADOLC and AD-Recorder) to make an OpenSim function *F* and its forward (*F fwd*) and reverse (*F rev*) directional derivatives available within the CasADi environment for use by the NLP solver during the optimization. In the AD-ADOLC approach (top), ADOL-C’s algorithms are used in a C++ code to provide *F fwd* and *F rev*. In the AD-Recorder approach (bottom), Recorder provides the expression graph of *F* as MATLAB source code from which CasADi’s C-code generator generates C-code containing *F*, *F fwd*, and *F rev*. The AD-Recorder approach combines operator overloading, when generating the expression graph, and source code transformation, when processing the expression graph to generate C-code for *F*, *F fwd*, and *F rev*. In both approaches, the code comprising *F*, *F fwd*, and *F rev* is compiled as a Dynamic-link Library (DLL), which is imported as an external function within the CasADi environment. In our application, *F* represents the multi-body dynamics and is called when formulating the optimal control problem. The latter is then composed into a differentiable optimal control transcription using CasADi. During the optimization, CasADi provides the NLP solver with evaluations of the NLP objective function (*nlp f*), constraints (*nlp g*), objective function gradient (*nlp grad f*), constraint Jacobian (*nlp jac g*), and Hessian of the Lagrangian (*nlp hess l*). CasADi efficiently queries *F fwd* and *F rev* to construct the full derivative matrices.

We first incorporated the operator overloading AD tool ADOL-C in OpenSim. ADOL-C relies on the concept of active variables, which are variables that may be considered as differentiable quantities at some time during the execution of a computer program [18]. To distinguish these variables and store information about their partial derivatives, ADOL-C introduced the augmented scalar type *adouble* whose real part is of standard type *double*. All active variables should be of type *adouble*. To differentiate OpenSim functions using ADOL-C, we modified OpenSim’s source code by replacing the type of potential active variables to *adouble* (example for SimTK::square() in Fig 2). We maintained a layer of indirection so that OpenSim could be compiled to use either *double* or *adouble* as the scalar type. We excluded parts of the code, such as numerical optimizers, that were not relevant to this study.

The limited computational benefits of using AD through ADOL-C led us to seek alternative AD strategies (see discussion for more detail). We developed our own tool, Recorder, which combines the versatility of operator overloading and the speed of source code transformation. Recorder is a C++ scalar type for which all operators are overloaded to generate an expression graph. When evaluating an OpenSim function numerically at a nominal point, Recorder generates the function’s expression graph as MATLAB source code in a format that CasADi’s AD algorithms can transform into C-code (see S1 Appendix for source code from the example of Fig 1). Note that this workflow is currently only practical when the branches (*if-tests*) encountered at the nominal point remain valid for all evaluations encountered during the optimization.

To use Recorder with OpenSim, we relied on the code we had modified for incorporating ADOL-C but replaced *adouble* with the *Recorder* scalar type (example for SimTK::square() in Fig 2). This change required minimal effort but enabled Recorder to identify all differentiable variables when constructing the expression graphs.

### Interface between OpenSim and CasADi

We enabled the use of OpenSim functions within the CasADi environment by compiling the functions and their derivatives as Dynamic-link Libraries that are then imported as external functions for use by CasADi (Fig 2). The function derivatives can be computed through ADOL-C (AD-ADOLC in Fig 2) or through Recorder (AD-Recorder in Fig 2).

### Trajectory optimization problems to evaluate computational choices

We designed three example trajectory optimization problems to evaluate different computational choices (see Tables 1–3 for detailed formulations). The general formulation of the optimal control problems consists of computing the controls ***u***(*t*), states ***x***(*t*), and time-independent parameters ***p*** minimizing an objective functional:

$$J=\int_{t_{i}}^{t_{f}}L(x(t),u(t),p)dt,$$ (1)

where *t*_*i*_ and *t*_*f*_ are initial and final times, and *t* is time [37]. This objective functional is subject to dynamic constraints:

$$\dot{x}(t)=f(x(t),u(t),p),$$ (2)

and to algebraic path constraints:

$$g_{min}\leq g(x(t),u(t),p)\leq g_{max},$$ (3)

which are equality constraints if *g*_min_ = *g*_max_. The optimization variables are typically bounded as follows:

$$x_{min}\leq x(t)\leq x_{max},$$ (4)

$$u_{min}\leq u(t)\leq u_{max},$$ (5)

$$p_{min}\leq p\leq p_{max}.$$ (6)

**Table 1:** Formulation of example 1.

Pendulum simulations			
**Number of** **optimization variables**	2 degree of freedom pendulum: 504 3 degree of freedom pendulum: 756 4 degree of freedom pendulum: 1008 5 degree of freedom pendulum: 1260 6 degree of freedom pendulum: 1512 7 degree of freedom pendulum: 3514 8 degree of freedom pendulum: 4016 9 degree of freedom pendulum: 4518 10 degree of freedom pendulum: 10020	**Number of equality constraints**	2 degree of freedom pendulum: 458 3 degree of freedom pendulum: 687 4 degree of freedom: pendulum 916 5 degree of freedom pendulum: 1145 6 degree of freedom pendulum: 1374 7 degree of freedom pendulum: 3178 8 degree of freedom pendulum: 3632 9 degree of freedom pendulum: 4086 10 degree of freedom pendulum: 9040
**States** ***x***(*t*)	Joint positions *q* and velocities *v*	**Controls** ***u***(*t*)	Derivatives of *v* (accelerations): *u*_*dv*_ Joint torques *u*_*T*_
**Bounds**	$-3\pi =q_{lb}\leq q\leq q_{ub}=3\pi$ $-20=v_{lb}\leq v\leq v_{ub}=20$ $-500=u_{dv,lb}\leq u_{dv}\leq u_{dv,ub}=500$ $-1000=u_{T,lb}\leq u_{T}\leq u_{T,ub}=1000$	**Scaling**	$s_{q}=3;\;\tilde{q}=q/s_{q};\;{\tilde{q}}_{lb}=q_{lb}/s_{q};\;{\tilde{q}}_{ub}=q_{ub}/s_{q}$ $s_{t}=0.2;\;\tilde{v}=v/{(s}_{q}/s_{t});\;{\tilde{v}}_{lb}=v_{lb}/\left(s_{q}/s_{t}\right);\;{\tilde{v}}_{ub}=v_{ub}/(s_{q}/s_{t})$ ${\tilde{u}}_{dv}=u_{dv}/(s_{q}/{s_{t}}^{2});\;{\tilde{u}}_{dv,lb}=u_{dv,lb}/(s_{q}/{s_{t}}^{2});\;u_{dv,ub}=u_{dv,ub}/(s_{q}/{s_{t}}^{2})$ $s_{T}=500;\;{\tilde{u}}_{T}=u_{T}/s_{T};\;{\tilde{u}}_{T,lb}=u_{T,lb}/s_{T};\;{\tilde{u}}_{T,ub}=u_{T,ub}/s_{T}$
**Objective** **function**	$L={\left\|{\tilde{u}}_{T}\right\|}_{2}^{2}+L_{p}$ $L_{p}=0.1{\left\|{\tilde{u}}_{dv}\right\|}_{2}^{2}$	**Dynamic constraints**	$(dq⁄dt)/s_{q}=v/s_{q}$ $\left(dv/dt\right)/(s_{q}/s_{t})=u_{dv}/(s_{q}/s_{t})$
**Path** **constraints**	$T=f_{s}(q,v,u_{dv})$ $T/s_{T}={\tilde{u}}_{T}$ $\tilde{q}(0)=\tilde{q}(1)=\tilde{v}(0)=\tilde{v}(1)=0$		

**Controls**: we introduced accelerations (time derivative of velocities) as controls (implicit formulations) in addition to joint torques. **Bounds**: *lb* and *ub* are for lower and upper bounds, respectively. **Scaling**: we used time scaling for the joint states and controls. **Objective function**: to avoid singular arcs, situations for which controls are not uniquely defined by the optimality conditions [37], we appended a penalty function *L*_*p*_ with the remaining controls to the objective function *L*. **Dynamic constraints** are scaled using the same scale factors as used for the states [37]. We used implicit formulations. **Path constraints**: *f*_*s*_(∙) computes net joint torques *T* according to the skeleton dynamics.

**Table 2:** Formulation of example 2.

2D predictive simulations of walking			
**Number of** **optimization variables**	13807	**Number of** **constraints**	12857 equality constraints, 1800 inequality constraints
**States** ***x***(*t*)	Muscle activations *a* and tendon forces *F*_*t*_ Joint positions *q* and velocities *v* Trunk activations *a*_trunk_	**Controls** ***u***(*t*)	Derivatives of *a*: *u*_*da*_ and *F*_*t*_: $u_{dF_{t}}$ Derivatives of *v* (accelerations): *u*_*dv*_ Trunk excitations *e*_trunk_
**Parameters** ***p***	Half gait cycle duration *t*_*f*_	**Scaling**	$s_{q}=max(abs(q_{lb}),abs(q_{ub}));\;\tilde{q}=q/s_{q}$; ${\tilde{q}}_{lb}=q_{lb}/s_{q}$; ${\tilde{q}}_{ub}=q_{ub}/s_{q}$ $s_{v}=max(abs(v_{lb}),abs(v_{ub}));\;\tilde{v}=v/s_{v}$; ${\tilde{v}}_{lb}=v_{lb}/s_{v}$; ${\tilde{v}}_{ub}=v_{ub}/s_{v}$ $s_{u_{dv}}=max\left(abs(u_{dv,lb}),abs(u_{dv,ub})\right);\;{\tilde{u}}_{dv}=u_{dv}/s_{u_{dv}}$ ${\tilde{u}}_{dv,lb}=u_{dv,lb}/s_{u_{dv}}$; ${\tilde{u}}_{dv,ub}=u_{dv,ub}/s_{u_{dv}}$ $s_{da}=s_{dF_{t}}=100;\;s_{trunk}=150$ $s_{F_{t}}=F_{t,ub};\;{\tilde{F}}_{t}=F_{t}/s_{F_{t}}$; ${\tilde{F}}_{t,ub}=F_{t,ub}/s_{F_{t}}$
**Bounds**	$0\leq a\leq 1;\;0\leq F_{t}\leq F_{t,ub}=5;\;0.1\leq t_{f}\leq 1$ $-\tau_{d}/100\leq u_{da}\leq \tau_{a}/100;\;\tau_{d}=60ms;\;\tau_{a}=15ms$ $-1\leq u_{dF_{t}},a_{trunk},e_{trunk}\leq 1$ $q_{lb,man}=q_{lb}\leq q\leq q_{ub}=q_{ub,man}$ ${v_{lb,man}=v}_{lb}\leq v\leq v_{ub}=v_{ub,man}$ ${u_{dv,lb,man}=u}_{dv,lb}\leq u_{dv}\leq u_{dv,ub}=u_{dv,ub,man}$		
**Objective** **function**	$L=\left(w_{1}{\left\|a\right\|}_{3}^{3}+w_{2}{\left\|e_{trunk}\right\|}_{2}^{2}+w_{3}{\left\|{\tilde{u}}_{dv}\right\|}_{2}^{2}+L_{p}\right)/d$ $L_{p}=0.001({\left\|u_{da}\right\|}_{2}^{2}+{\left\|u_{dF_{t}}\right\|}_{2}^{2})$	**Dynamic** **constraints**	$da/dt=s_{da}u_{da}$ $\left(dF_{t}/dt\right)/s_{F_{t}}=\left(s_{dF_{t}}u_{dF_{t}}\right)/s_{F_{t}}$ $\left(dq/dt\right)/s_{q}=v/s_{q}$ $\left(dv/dt\right)/s_{v}=u_{dv}/s_{v}$ $da_{trunk}/dt=\left(e_{trunk}-a_{trunk}\right)/\tau ;\;\tau =35ms$
**Path** **constraints**	$0\leq s_{da}u_{da}+a/\tau_{d}$ $s_{da}u_{da}+a/\tau_{a}\leq 1/\tau_{a}$ $f_{c}(a,F_{t},u_{dF_{t}})=0$ $T=f_{s}(q,v,u_{dv})$ $T_{pelvis}=0$	$T_{ll}=\sum_{m=1}^{M}{MA}_{m}F_{t,m}$ $T_{trunk}/s_{trunk}=a_{trunk}$ $\bar{x}(t_{f})=\bar{x}(0)$ $\left(q_{pelvis,for}(t_{f})-q_{pelvis,for}(0)\right)/t_{f}=1.33$	

**Controls** are introduced for the time derivative of the states (implicit formulations) in addition to trunk excitations. **Bounds** are manually (man) set for the joint states and controls; *lb* and *ub* are for lower and upper bounds, respectively. **Scaling**: joint states and controls, and tendon forces are scaled such that the lower and upper bounds are between -1 and 1. **Objective function** *L* is normalized by distance traveled *d*. To avoid singular arcs [37], a penalty function *L*_*p*_ (with low weight) with the remaining controls is appended to *L*. **Dynamic constraints** are scaled using the scale factors used for the states [37]. **Path constraints**: *f*_*s*_(∙) computes net joint torques *T* according to the skeleton dynamics, *f*_*c*_(∙) describes the Hill-type muscle contraction dynamics [10], *MA*_*m*_ is moment arm of muscle $m,\;\bar{x}(∙)$ contains all states except the pelvis forward position *q*_pelvis,for_ (symmetry), and 1.33 m s^-1^ is the prescribed gait speed.

**Table 3:** Formulation of example 3.

3D tracking simulations of walking			
**Number of optimization variables**	61318	**Number of constraints**	56050 equality constraints, 9200 inequality constraints
**States** ***x***(*t*)	Muscle activations *a* and tendon forces *F*_*t*_ Joint positions *q* and velocities *v* Arm activations *a*_arms_	**Controls** ***u***(*t*)	Derivatives of *a*: *u*_*da*_ and *F*_*t*_: $u_{dF_{t}}$ Derivatives of *v* (accelerations): *u*_*dv*_ Arm excitations *e*_arms_
**Parameters** ***p***	Contact sphere transversal plane locations *p*_*cl*_ Contact sphere radii *p*_*cr*_	**Scaling**	$s_{q}=max(abs(q_{lb}),abs(q_{ub}));\;\tilde{q}=q/s_{q};\;{\tilde{q}}_{lb}=q_{lb}/s_{q}$; ${\tilde{q}}_{ub}=q_{ub}/s_{q}$ $s_{v}=max(abs(v_{lb}),abs(v_{ub}));\;\tilde{v}=v/s_{v};\;{\tilde{v}}_{lb}=v_{lb}/s_{v}$; ${\tilde{v}}_{ub}=v_{ub}/s_{v}$ $s_{u_{dv}}=max(abs(u_{dv,lb}),abs(u_{dv,ub}));\;{\tilde{u}}_{dv}=u_{dv}/s_{u_{dv}}$ ${\tilde{u}}_{dv,lb}=u_{dv,lb}/s_{u_{dv}}$; ${\tilde{u}}_{dv,ub}=u_{dv,ub}/s_{u_{dv}}$ $s_{da}=s_{dF_{t}}=100;\;s_{T}=s_{arms}=150$ $s_{F_{t}}=F_{t,ub};\;{\tilde{F}}_{t}=F_{t}/s_{F_{t}}$; ${\tilde{F}}_{t,ub}=F_{t,ub}/s_{F_{t}}$ ${\tilde{p}}_{cl,v}=1/\left(p_{cl,ub}-p_{cl,lb}\right);\;{\tilde{p}}_{cl,r}=0.5-p_{cl,ub}/\left(p_{cl,ub}-p_{cl,lb}\right)$ ${\tilde{p}}_{cr,v}=1/\left(p_{cr,ub}-p_{cr,lb}\right);\;{\tilde{p}}_{cr,r}=0.5-p_{cr,ub}/\left(p_{cr,ub}-p_{cr,lb}\right)$ ${\tilde{p}}_{cl,lb}={\tilde{p}}_{cr,lb}=-0.5;\;{\tilde{p}}_{cl,ub}={\tilde{p}}_{cr,ub}=0.5$
**Bounds**	$0\leq a\leq 1;\;0\leq F_{t}\leq F_{t,ub}=5$ $-\tau_{d}/100\leq u_{da}\leq \tau_{a}/100;\;\tau_{d}=60ms;\;\tau_{a}=15ms$ $-1\leq u_{dF_{t}},a_{arms},e_{arms}\leq 1$ ${\hat{q}}_{min}-{\hat{q}}_{r}=q_{lb}\leq q\leq q_{ub}={\hat{q}}_{max}+{\hat{q}}_{r}$ ${\hat{v}}_{min}-{\hat{v}}_{r}=v_{lb}\leq v\leq v_{ub}={\hat{v}}_{max}+{\hat{v}}_{r}$ ${\hat{u}}_{dv,min}-{\hat{u}}_{dv,r}=u_{dv,lb}\leq u_{dv}\leq u_{dv,ub}={\hat{u}}_{dv,max}+{\hat{u}}_{dv,r}$ ${\hat{p}}_{ci}-0.025=p_{cl,lb}\leq p_{cl}\leq p_{cl,ub}={\hat{p}}_{ci}+0.025$ ${\hat{p}}_{cr}-0.5{\hat{p}}_{cr}=p_{cr,lb}\leq p_{cr}\leq p_{cr,ub}={\hat{p}}_{cr}+0.5{\hat{p}}_{cr}$		
**Objective** **function**	$L=w_{1}{\left\|a\right\|}_{2}^{2}+w_{2}{\left\|q-\hat{q}\right\|}_{2}^{2}+w_{3}{\left\|GRF-\hat{GRF}\right\|}_{2}^{2}+w_{4}{\left\|GRT-\hat{GRT}\right\|}_{2}^{2}+w_{5}{\left\|T_{ll,trunk,arms}-{\hat{T}}_{ll,trunk,arms}\right\|}_{2}^{2}+L_{p}$ $L_{p}=0.001\;({\left\|u_{da}\right\|}_{2}^{2}+{\left\|u_{dF_{t}}\right\|}_{2}^{2}+{\left\|u_{dv}\right\|}_{2}^{2})$	**Dynamic constraints**	$da/dt=s_{da}u_{da}$ $\left(dF_{t}/dt\right)/s_{F_{t}}=\left(s_{dF_{t}}u_{dF_{t}}\right)/s_{F_{t}}$ $\left(dq/dt\right)/s_{q}=v/s_{q}$ $\left(dv/dt\right)/s_{v}=u_{dv}/s_{v}$ $da_{arms}/dt=\left(e_{arms}-a_{arms}\right)/\tau ;\;\tau =35ms$
**Path** **constraints**	$0\leq s_{da}u_{da}+a/\tau_{d}$ $s_{da}u_{da}+a/\tau_{a}\leq 1/\tau_{a}$ $f_{c}(a,F_{t},u_{dF_{t}})=0$ $T=f_{s}(q,v,u_{dv})$	$T_{pelvis}/s_{T}={\hat{T}}_{pelvis}/s_{T}$ $T_{ll,trunk}=\sum_{m=1}^{M}{MA}_{m}F_{t,m}+T_{p}$ $T_{arms}/s_{arms}=a_{arms}$	

**Controls** are introduced for the state derivatives in addition to arm excitations. **Bounds** of joint states and controls are based on measured data ($\hat{q},\;\hat{u},\;{\hat{u}}_{dv}$); min and max are for minimum and maximum values, respectively; *r* is range of motion; *lb* and *ub* are for lower and upper bounds, respectively; bounds of contact parameters (*p*_*cl*_, *p*_*cr*_) are based on [4]. **Scaling**: joint states and controls, and tendon forces are scaled such that the lower and upper bounds are between -1 and 1; contact parameters are scaled such that their lower and upper bounds are -0.5 and 0.5, respectively. **Objective function** *L* tracks measured joint positions ($\hat{q}$), ground reaction forces ($\hat{GRF}$) and torques ($\hat{GRT}$), and joint torques of the lower limbs, trunk, and arms (${\hat{T}}_{ll,trunk,arms}$). A penalty function *L*_*p*_ is appended to *L*. **Dynamic constraints** are scaled using scale factors used for the states [37]. **Path constraints**: *f*_*s*_(∙) computes net joint torques *T* according to the skeleton dynamics, *f*_*c*_(∙) describes the Hill-type muscle contraction dynamics [10], *MA*_*m*_ is moment arm of muscle *m*, and *T*_*p*_ are passive torques [5].

In the first example, we perturbed the balance of nine inverted pendulums, with between two and 10 degrees of freedom, by applying a backward translation to their base of support. The optimal control problem identified the joint torques necessary to restore the pendulums’ upright posture within one second while minimizing the actuator effort (i.e., squared joint torques) and satisfying the pendulum dynamics.

In the second example, we performed predictive simulations of walking with a two-dimensional (2D) musculoskeletal model (10 degrees of freedom, 18 muscles actuating the lower limbs, one ideal torque actuator at the trunk, and two contact spheres per foot [24]). We identified muscle excitations and half walking cycle duration that minimized a weighted sum of muscle fatigue (i.e., muscle activations at the third power [6]) and joint accelerations subject to constraints describing the musculoskeletal dynamics, imposing left-right symmetry, and prescribing gait speed (i.e., distance travelled by the pelvis divided by gait cycle duration). Imposing left-right symmetry allowed us to only optimize for half a gait cycle.

In the third example, we performed tracking simulations of walking with a three-dimensional (3D) musculoskeletal model (29 degrees of freedom, 92 muscles actuating the lower limbs and trunk, eight ideal torque actuators at the arms, and six contact spheres per foot [4,24,38]) while calibrating the foot-ground contact model. We identified muscle excitations and contact sphere parameters (locations and radii) that minimized a weighted sum of muscle effort (i.e., squared muscle activations) and the difference between measured and simulated variables (joint angles and torques, and ground reaction forces and torques) while satisfying the musculoskeletal dynamics. Data collection was approved by the Ethics Committee at UZ / KU Leuven (Belgium).

In these examples, we modeled pendulum/skeletal movement with Newtonian rigid body dynamics and, for the walking simulations, compliant Hunt-Crossley foot-ground contact [24,26]. We created a continuous approximation of a contact model from Simbody to provide twice continuously differentiable contact forces, which are required when using second-order gradient-based optimization algorithms [39]. We performed the approximations of conditional *if-tests* using hyperbolic tangent functions. For the muscle-driven walking simulations, we described muscle activation and contraction dynamics using Raasch’s model [9,40] and a Hill-type muscle model [10,41], respectively. We defined muscle-tendon lengths, velocities, and moment arms as a function of joint positions and velocities using polynomial functions [42]. We optimized the polynomial coefficients to fit muscle-tendon lengths and moment arms (maximal root mean square deviation: 3 mm; maximal order: ninth) obtained from OpenSim for a wide range of joint positions.

We transcribed each optimal control problem into a NLP using a third order Radau quadrature collocation scheme. We formulated each problem in MATLAB using CasADi and IPOPT. We imposed an NLP relative error tolerance of 1 x 10^−6^ and used an adaptive barrier parameter update strategy. We selected a number of mesh intervals for each problem such that the results were qualitatively similar when using a mesh twice as fine. We used 10 and three initial guesses for the pendulum and walking simulations, respectively. We ran all simulations on a single core of a standard laptop computer with a 2.9 GHz Intel Core i7 processor.

### Results analysis

We compared CPU time and number of iterations required to solve the problems using the different computational choices. First, we compared AD, using the Recorder approach, with FD. Second, we compared the AD approaches, namely AD-Recorder and AD-ADOLC. We performed these two comparisons using the linear solver mumps [43], which CasADi provides, and an approximated Hessian. Third, we compared different linear solvers, namely mumps with the collection of solvers from HSL (ma27, ma57, ma77, ma86, and ma97) [44], while using AD-Recorder and an approximated Hessian. Finally, we compared the use of approximated and exact Hessians. For this last comparison, we used AD-Recorder and tested all linear solvers. In all cases, we ran simulations from different initial guesses and compared results from simulations that started from the same initial guess and converged to similar optimal solutions. Table 4 distinguishes the numerical tools used in our analyses.

**Table 4:** Numerical tools.

NLP solver	Linear solvers		AD approaches
IPOPT	Mumps		Operator overloading (ADOL-C)
	HSL collection	ma27 ma57 ma77 ma86 ma97	
			Source code transformation (Recorder)

## Results

**Fig 3:**
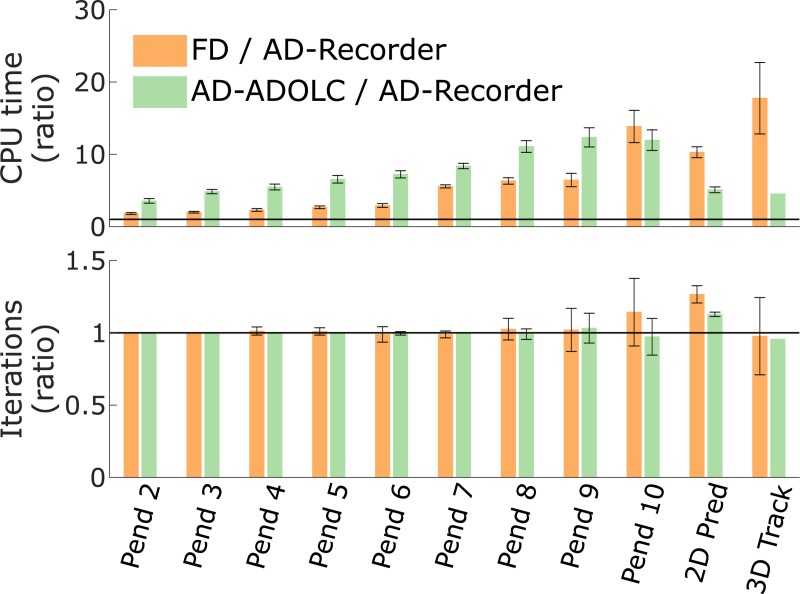
**Comparison of computational time (top) and number of iterations (bottom) between FD, AD-ADOLC, and AD-Recorder.** The comparisons are expressed as ratios and averaged over results from different initial guesses (error bars represent ± one standard deviation). The horizontal lines indicate 1:1 ratios. Ratios larger than one indicate slower convergence (top) and more iterations (bottom) with FD or AD-ADOLC as compared to AD-Recorder. Pend indicates pendulum simulations with the number being the number of degrees of freedom; Pred and Track indicate predictive and tracking simulations, respectively. The results were obtained using mumps and an approximated Hessian.

Using AD-Recorder was computationally more efficient than using FD or AD-ADOLC (Fig 3). The CPU time decreased when using AD-Recorder as compared to FD (between 1.8 ± 0.1 and 17.8 ± 4.9 times faster with AD-Recorder) and AD-ADOLC (between 3.6 ± 0.3 and 12.3 ± 1.3 times faster with AD-Recorder). CPU time spent in evaluating the objective function gradient accounted for 95 ± 10% (average ± standard deviation) of the difference in CPU time between AD-Recorder and FD. The difference in CPU time spent in evaluating the constraint Jacobian accounted for 89 ± 6% of the difference in CPU time between AD-Recorder and AD-ADOLC. The number of iterations was similar when using AD-Recorder, FD, and AD-ADOLC. For the 2D predictive and 3D tracking simulations, one and two cases, respectively, out of nine (three derivative scenarios and three initial guesses) were excluded from the comparison as they converged to different solutions.

The solvers from the HSL collection were on average more efficient (faster with a similar number of iterations) than mumps for the pendulum simulations, but the efficiency varied for the 2D predictive and 3D tracking simulations (Table 5). The solver ma27 was on average faster than mumps in all cases although ma27 required more iterations for the 2D predictive simulations. The other solvers from the HSL collection were on average slower than mumps for the 2D predictive simulations. For the 3D tracking simulations, the solvers ma77 and ma86 were faster and slower, respectively, than mumps. The solvers ma57 and ma97 failed to solve the 3D tracking simulations due to memory issues. For all simulations, the solvers from the HSL collection except ma86 (and ma77 for the 2D predictive simulations) required less CPU time per iteration than mumps. For the 2D predictive and 3D tracking simulations, one case out of 18 (six solvers and three initial guesses) and four cases out of 12 (four solvers and three initial guesses), respectively, were excluded from the comparison as they converged to different solutions.

**Table 5:** Comparison of computational time, number of iterations, and computational time per iteration between linear solvers.

Solver * vs mumps	Pendulum simulations			2D predictive simulations			3D tracking simulations		
	CPU time	Iteration Number	CPU time per Iteration	CPU time	Iteration Number	CPU time per Iteration	CPU time	Iteration Number	CPU time per Iteration
*ma27	0.6 ± 0.1	1.0 ± 0.0	0.6 ± 0.1	1.0 ± 0.3	1.4 ± 0.3	0.7 ± 0.1	0.7 ± 0.2	0.9 ± 0.3	0.7 ± 0.0
*ma57	0.6 ± 0.0	1.0 ± 0.0	0.6 ± 0.0	2.4 ± 2.5	2.8 ± 2.8	0.8 ± 0.0	/	/	/
*ma77	0.9 ± 0.1	1.0 ± 0.0	0.9 ± 0.0	1.3 ± 0.0	1.2 ± 0.0	1.1 ± 0.0	0.5 ± 0.2	0.7 ± 0.3	0.7 ± 0.0
*ma86	1.1 ± 0.1	1.0 ± 0.0	1.1 ± 0.1	2.1 ± 0.5	1.1 ± 0.1	1.8 ± 0.3	2.3 ± 0.0	1.9 ± 0.0	1.2 ± 0.0
*ma97	0.7 ± 0.0	1.0 ± 0.0	0.7 ± 0.0	1.2 ± 0.3	1.2 ± 0.1	1.0 ± 0.2	/	/	/

The comparisons are expressed as ratios (mean ± one standard deviation; results obtained with solver from the HSL collection over results obtained with mumps

* indicates ma27, ma57, ma77, ma86, or ma97).

The ratios are averaged over results from different initial guesses. Ratios larger than one indicate faster convergence, fewer iterations, or less time per iteration with mumps. The use of the solvers ma57 and ma97 led to memory issues for the 3D tracking simulations and these cases were therefore excluded from the analysis. The simulations were run using AD-Recorder and an approximated Hessian.

**Fig 4:**
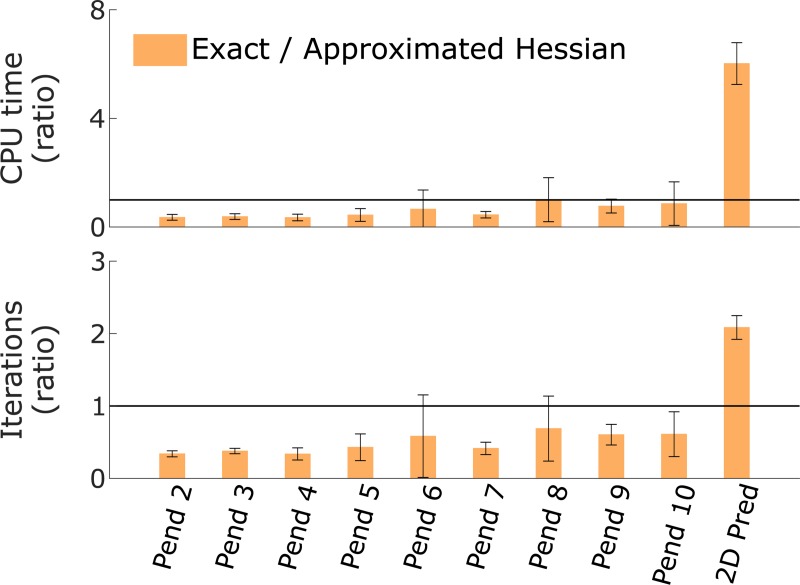
**Comparison of computational time (top) and number of iterations (bottom) between exact and approximated Hessian.** The comparisons are expressed as ratios and averaged over results from different initial guesses (error bars represent ± one standard deviation). The horizontal lines indicate 1:1 ratios. Ratios larger than one indicate slower convergence (top) and more iterations (bottom) with an exact versus an approximated Hessian. Pend indicates pendulum simulations with the number being the number of degrees of freedom; Pred and Track indicate predictive and tracking simulations, respectively. The results were obtained using all solvers and AD-Recorder.

Using an exact Hessian was more efficient than using an approximated Hessian for the pendulum simulations but not for the 2D predictive simulations (Fig 4). The exact Hessian required less CPU time and fewer iterations than the approximated Hessian for the pendulum simulations (average 2.4 ± 1.2 times faster and 2.5 ± 0.9 times fewer iterations). By contrast, the exact Hessian required more CPU time and iterations than the approximated Hessian for the 2D predictive simulations (average 6.0 ± 0.8 times slower and 2.1 ± 0.2 times more iterations). For the pendulum simulations, 27 cases out of 540 (nine pendulums, six solvers, and 10 initial guesses) were excluded from the comparison as they converged to different solutions with the two Hessian settings. One case was also excluded as it had not converged after 3000 iterations with the exact Hessian but converged in 209 iterations with the approximated Hessian. For the 2D predictive simulations, only results obtained with the solvers ma86 and ma97 were included, since the use of the other solvers led to memory issues. Further, four cases out of six (two solvers and three initial guesses) were excluded from the comparison as they converged to different solutions with the two Hessian settings. Finally, the 3D tracking simulations were not included for this comparison as the large problem size induced memory issues with the exact Hessian.

In the different analyses, we examined the cases that we excluded from the comparison because of convergence to different solutions but we did not find that one derivative scenario, solver, or initial guess consistency led to a local optimum with a lower cost.

The pendulum simulations required at most 21 s and 366 iterations to converge (results obtained with AD-Recorder, mumps, and an approximated Hessian); CPU time and number of iterations depended on the number of degrees of freedom (S1 Movie).

**Fig 5:**
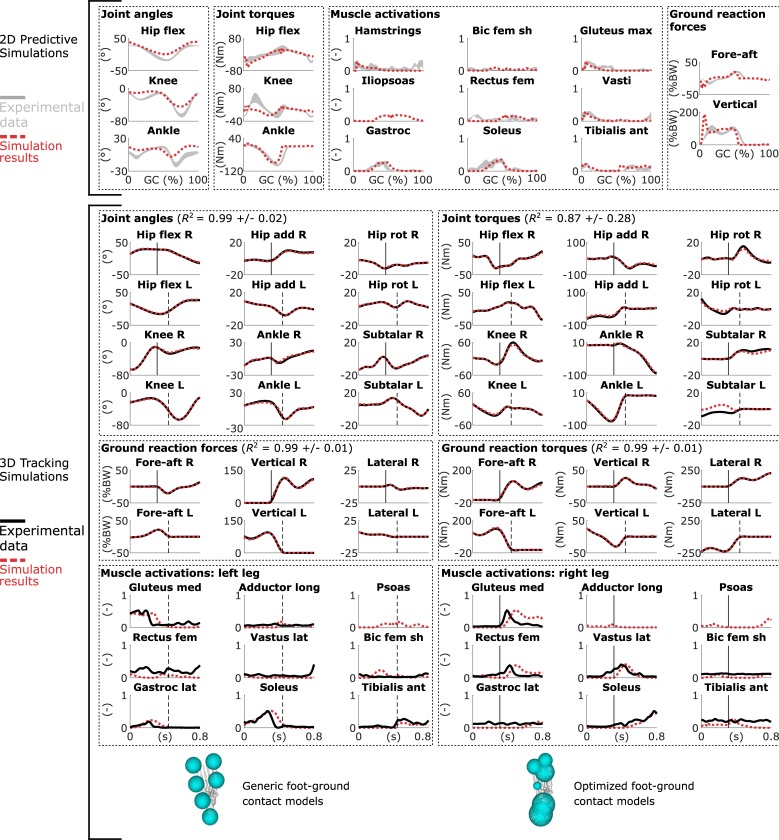
Results from trajectory optimization of walking. (Top) Results from 2D predictive simulations of walking (joint angles: flex is flexion, GC is gait cycle; muscle activations: bic is biceps, fem is femoris, sh is short head, max is maximus, gastroc is gastrocnemius, ant is anterior; ground reaction forces: BW is body weight). Experimental data are shown as mean ± two standard deviations. (Bottom) Results from 3D tracking simulations of walking (joint angles: R is right, L is left, add is adduction, rot is rotation; muscle activations: med is medialis, long is longus, lat is lateralis). The vertical lines indicate right heel strike (solid) and left toe-off (dashed); only part of the gait cycle, when experimental ground reaction forces are available, is tracked. The experimental electromyography data is normalized to peak muscle activations. The foot diagrams depict a down-up view of the configuration of the contact spheres of the right foot pre-calibration (left: generic) and post-calibration (right: optimized). The coefficient of determination *R*^*2*^ is given for the tracked variables.

The 2D predictive simulations reproduced salient features of human gait but deviated from experimental data in three noticeable ways (Fig 5; S2 Movie). First, the predicted knee flexion during mid-stance was limited, resulting in small knee torques. Second, the simulations produced less ankle plantarflexion at push-off. Third, the vertical ground reaction forces exhibited a large peak at impact. The simulations converged in less than one CPU minute (average over solutions starting from three initial guesses: 36 ± 17 s and 247 ± 143 iterations; results obtained with AD-Recorder, mumps, and an approximated Hessian).

The 3D tracking simulations accurately tracked the experimental walking data (average coefficient of determination *R*^*2*^: 0.95 ± 0.17; Fig 5; S3 Movie). Simulated muscle activations also qualitatively resembled experimental electromyography data, even though electromyography was not tracked (Fig 5). The configuration of the contact spheres differed from the generic model after the calibration. The simulations converged in less than 20 CPU minutes (average over simulations starting from two initial guesses: 19 ± 7 minutes and 493 ± 151 iterations; results obtained with AD-Recorder, mumps, and an approximated Hessian).

## Discussion

We showed that the use of AD over FD improved the computational efficiency of OpenSim-based trajectory optimization of human movement. Specifically, AD drastically decreased the CPU time spent in evaluating the objective function gradient. This time decrease results from AD’s ability to evaluate a Jacobian-transposed-times-vector product through its reverse mode. The objective function gradient has many inputs (all optimization variables) but only one output. It can thus be evaluated in only one reverse sensitivity sweep; the computational cost is hence proportional to the cost of evaluating the objective function. By contrast, with FD, the computational cost is proportional to the number of optimization variables times the cost of evaluating the objective function. The efficiency benefit of AD also increased with the complexity of the problems. This is expected, since the number of optimization variables increases with problem size; FD thus requires more objective function evaluations, whereas AD still requires only one reverse sweep. In our problems, AD did not outperform FD when evaluating the constraint Jacobian. Yet we expect that AD will be more efficient than FD for trajectory optimization problems in which the number of optimization variables largely exceeds the number of constraints, thereby resulting in faster constraint Jacobian evaluations with AD’s reverse mode.

The choice of the objective function influences CPU time. As an illustration, we added a term representing the metabolic energy rate [45] to the objective function of the 2D predictive simulations. Minimizing metabolic energy rate is common in predictive studies of walking [5,7,39]. Solving the resulting optimal control problem was about 60 times faster with AD-Recorder than with FD (although FD required fewer iterations), whereas AD-Recorder was only about 10 times faster than FD without incorporating the metabolic energy rate in the objective function. This increased time difference can be explained by our use of computationally expensive hyperbolic tangent functions to make the metabolic energy rate model twice continuously differentiable, as required when using second-order gradient-based optimization algorithms [39]. Overall, AD reduces the number of function evaluations, which has an even larger effect if these functions are expensive to compute.

The implementation of AD was computationally more efficient through Recorder than through ADOL-C. Specifically, Recorder decreased the CPU time by a factor 4–12 compared to ADOL-C. ADOL-C records all calculations involving differential variables on a sequential data set called a tape [18], which is then evaluated by ADOL-C’s *virtual machine*. By contrast, Recorder generates plain C-code. The factor 4–12 is the difference between a *virtual machine* interpreting a list of instructions (ADOL-C) and machine code performing these instructions directly (Recorder).

The effort required to enable the use of AD through Recorder was minimal once OpenSim’s source code had been modified for use with the ADOL-C libraries. Indeed, Recorder relies on operator overloading for constructing the expression graphs, which is similar to ADOL-C. The only required change was to replace the *adouble* scalar type (ADOL-C) by the *Recorder* scalar type. Recorder also facilitates the interface with CasADi, since it generates expression graphs in a format from which CasADi can directly generate C-code. This code can then be compiled as a Dynamic-link Library and imported in the CasADi environment without any scripting input required from the user (Fig 2). Using ADOL-C’s AD algorithms with CasADi necessitates manually writing C++ code to provide forward and reverse directional derivatives using ADOL-C’s drivers in a format recognized by CasADi, which might be prone to errors (Fig 2). Note that the manual effort required for using Recorder or ADOL-C is independent of problem complexity. Overall, using Recorder is more efficient but also simpler than using ADOL-C when solving trajectory optimization problems with CasADi.

The process of converting OpenSim’s source code to code that compiles with the AD tools (ADOL-C and Recorder) was a considerable but one-time effort. OpenSim-based trajectory optimization problems can now be solved through the proposed framework while benefiting from AD and without any additional developments. We made our OpenSim-based AD framework available so that others can build upon our work. Importantly, using AD does not increase the complexity for the end user as compared to using FD. Indeed, the simulation framework relies on CasADi that provides evaluations of function derivatives to the NLP solver. Hence, the user does not need to re-implement AD’s forward and reverse algorithms. It is also worth mentioning that, in this study, we used Recorder to enable the use of AD with OpenSim. However, Recorder is a general C++ class that could be applied to any other C++ code for use with CasADi. Compiling existing source code with Recorder would require replacing the scalar type of active variables (i.e., differentiable quantities) with the *Recorder* scalar type. Our study suggests that this programming effort might be particularly valuable when the goal is to solve complex trajectory optimization problems. Specifically, our results showed that the difference between AD and FD increased with problem size. Users might thus consider the programming effort only when the aim is to solve multiple complex problems and when they are not satisfied with the computational performance obtained with FD.

It is difficult to provide guidelines for the linear solver selection based on our results, as their efficiency was problem-dependent. In contrast with mumps, the solvers from the HSL collection do not freely come with CasADi and are only free to academics. Hence, our study does not support the extra effort to obtain them since they did not consistently outperform mumps in our applications. Yet an in-depth analysis of the solvers’ options and underlying mathematical details should be considered in future work.

The use of an exact Hessian, rather than an approximated Hessian, improved the computational efficiency for the pendulum simulations but not for the walking simulations. For the 2D walking simulations, using an exact Hessian required more CPU time but also more iterations. This might seem surprising, since an exact Hessian is expected to provide more accurate information and, therefore, lead to convergence in fewer iterations. However, IPOPT requires the Hessian to be positive definite when calculating a Newton step to guarantee that the step is in the descent direction. When this is not the case, the Hessian is approximated with a positive definite Hessian by adding the identity matrix multiplied by a regularization term to the Hessian [36]. We observed that for the 2D predictive simulations, the magnitude of the regularization term was much greater than for the pendulum simulations. Yet excessive regularization might degrade the performance of the algorithm, as regularization alters the second-order derivative information and causes IPOPT to behave more like a steepest-descent algorithm [46]. The approximated Hessian requires no regularization, which likely explains the difference in number of iterations. Overall, convexification of the currently non-convex optimal control problems is expected to further improve the computational efficiency [9].

Our comparison of derivative scenarios (AD-ADOLC, AD-Recorder, and FD), linear solvers (mumps and the HSL collection), and Hessian calculation schemes was based on several specific choices. First, we solved all problems using the NLP solver IPOPT, whereas other solvers compatible with CasADi, such as SNOPT [47] and KNITRO [48] (see [21] for detailed list), might behave differently. We selected IPOPT since it is open-source (SNOPT and KNITRO are commercial products), widely used, and well suited for large and very sparse NLPs [21]. Second, we transcribed the optimal control problems into NLPs using a third order Radau quadrature collocation scheme, whereas different orders, schemes (e.g., Legendre), and transcription methods (e.g., trapezoidal and Hermite-Simpson) might lead to different results. We selected quadrature collocation methods as they achieve exponential convergence if the underlying function is sufficiently smooth [1,49]. Third, we used specific models of muscle activation dynamics, contraction dynamics, and compliant contacts, whereas other models might behave differently. We selected models that were continuously differentiable for use with gradient-based optimization algorithms. Finally, our focus was on solving trajectory optimization problems for biomechanical applications with OpenSim. We chose OpenSim as it is an open-source and widely used software package in biomechanics. The difference in computational performance between AD and FD might thus vary with other software packages and applications. Investigating all these other modeling and computational choices was out of the scope of this study but might be useful for helping users select the best settings for their applications. Overall, our study underlined the computational benefit of using AD over FD for trajectory optimization in biomechanics, which is in agreement with previous research in robotics (e.g., [27]).

The 2D predictive and 3D tracking simulations produced realistic movements although deviations remain between simulated and measured data. Modeling choices rather than local optima likely explain these deviations. These choices have a greater influence on the predictive simulations, since deviations from measured data are minimized in tracking simulations, whereas only the motor task goal is specified in the objective function of predictive simulations. Several modeling choices might explain the main deviations for the predictive simulations. First, we did not model stability requirements, which might explain the limited knee flexion during mid-stance [6,39]. Instead, we included muscle activity in the cost function, which might explain why reducing knee torques and, therefore, knee extensor activity was optimal. Second, the model did not include a metatarsophalangeal joint, which might explain the limited ankle plantarflexion at push-off; similar ankle kinematics have indeed been observed experimentally when limiting the range of motion of the metatarsophalangeal joint [50]. Third, the lack of knee flexion combined with the simple trunk model (i.e., one degree of freedom controlled by one ideal torque actuator) might explain the high vertical ground reaction forces at impact [6]. Finally, the goal of the motor task (i.e., minimizing muscle fatigue) likely does not fully explain the control strategies governing human walking. In this study, the focus was on evaluating different computational choices but future work should exploit the improved computational efficiency to explore how modeling choices affect the correspondence between simulated and measured quantities.

Our results indicate that AD is particularly beneficial with increasingly complex models. Hence, our OpenSim-based AD framework might allow researchers to rely on complex models, such as three-dimensional muscle-driven neuro-musculoskeletal models, in their studies. This model complexity might be highly desirable when studying, for instance, the impact of treatment on gait performance in patients with neuro-musculoskeletal disorders. Indeed, in such cases, the model should be complex enough to describe the musculoskeletal structures and motor control processes underlying gait that may be affected by treatment. Previous studies based on predictive models reported high computational times and were therefore limited to few predictions when relying on complex musculoskeletal models [5,8,51]. Using AD has the potential to drastically decrease the computational time of such predictive simulations, thereby extending their application.

## Conclusions

In this study, we enabled the use of AD when performing OpenSim-based trajectory optimization of human movement. We showed that using AD drastically improved the computational efficiency of such simulations. This improved efficiency is highly desirable for researchers using complex models or aiming to implement such models in clinical practice where time constraints are typically more stringent than in research context. Overall, the combination of AD with other efficient numerical tools such as direct collocation and implicit differential equations allows overcoming the computational roadblocks that have long limited the use of trajectory optimization for biomechanical applications. In the future, we aim to exploit this computational efficiency to design optimal treatments for neuro-musculoskeletal disorders, such as cerebral palsy.

## Supporting information

S1 Appendix Example source code. Recorder provides the expression graph of the function to differentiate as MATLAB source code in a format that CasADi’s AD algorithms can then transform into C-code. This file provides MATLAB and C source code resulting from applying these two steps on the example function from Fig 1.
(PDF)

S1 Movie Pendulum-based simulations of perturbed balance. The pendulums have between two and 10 degrees of freedom. The playback speed is 0.2 times real-time.
(AVI)

S2 Movie 2D muscle-driven predictive simulation of walking (1.33 m s^-1^). The playback speed is 0.2 times real-time.
(MP4)

S3 Movie 3D muscle-driven tracking simulation of walking. The pink model (tracking simulation results) tracks the motion of the white model (experimental data). The playback speed is 0.2 times real-time.
(MP4)
