# Supplementary material for: Algorithmic differentiation improves the computational efficiency of OpenSim-based trajectory optimization of human movement
Source: PLoS One. 2019 Oct 17;14(10):e0217730. doi: 10.1371/journal.pone.0217730 (PMC6797126; doi:10.1371/journal.pone.0217730)
Supplement: S1 Appendix — Recorder provides the expression graph of the function to differentiate as MATLAB source code in a format that CasADi’s AD algorithms can then transform into C-code. This file provides MATLAB and C source code resulting from applying these two steps on the example function from Fig 1. (PDF) [file pone.0217730.s001.pdf]

Supplementary Material for: Algorithmic differentiation improves the computational efficiency of OpenSim-based trajectory optimization of human movement

Antoine Falisse, Gil Serrancolí, Christopher L. Dembia, Joris Gillis, and Friedl De Groote

PLoS ONE

Example source code

Figure 1 provides a flowchart of the expression graph of the function:

$$y = f(x_1, x_2) = \cos x_2 - x_2 x_1. \tag{1}$$

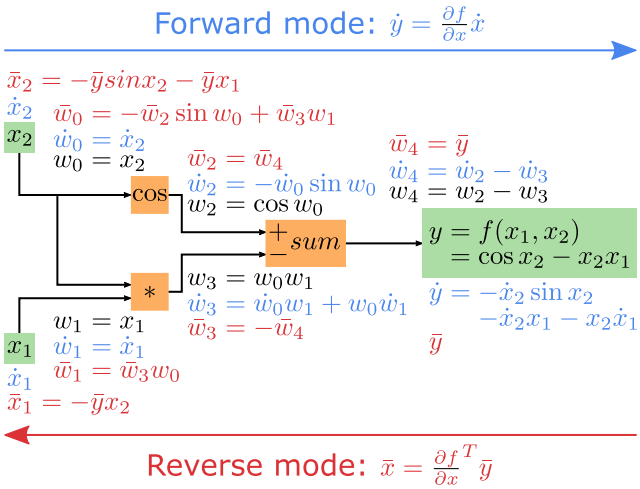

Figure 1: **Example of AD forward and reverse modes.** A function $y = f(x_1, x_2) = \cos x_2 - x_2 x_1$ is broken down into a sequence of elementary operations, forming an expression graph. In the forward mode, the forward seeds $\dot{x}_1$ and $\dot{x}_2$ are propagated from the inputs to the output and the Jacobian $J = \partial f / \partial \boldsymbol{x}$ relates $\dot{x}_1$ and $\dot{x}_2$ and forward sensitivity $\dot{y}$. In the reverse mode, the reverse seed $\bar{y}$ is propagated from the output to the inputs and the transposed Jacobian J^T relates $\bar{y}$ and reverse sensitivities $\bar{x}_1$ and $\bar{x}_2$.

When using the AD-Recorder approach, Recorder provides the expression graph of the function to differentiate as MATLAB source code. The following box provides MATLAB source code generated by Recorder upon evaluation of the function described in (1) with random inputs (here $x_2 = 2$ and $x_1 = 3$):

```
function [y]=foo(x)
nom = nargin==0;
if nom
  a1 = 2.0000000000000000e+00;
else
  a1 = x(1);
end
if nom
  a2 = 3.0000000000000000e+00;
else
  a2 = x(2);
end
a3 = times(a1,a2);
if nom, assert(a3==6.0000000000000000e+00); end;
a4 = cos(a1);
if nom, assert(a4==-4.1614683654714241e-01); end;
a5 = minus(a4,a3);
if nom, assert(a5==-6.4161468365471421e+00); end;
if ~nom
y{1} = a5;%-6.4161468365471421e+00
end
if ~nom, y = vertcat(y{:}); end;
```

The MATLAB source code is generated in a format from which CasADi's AD algorithms can then generate C-code. The following box provides C source code generated by CasADi. The code contains the function `F()` from (1) and its forward `fwd1_F()` and reverse `adj1_F()` directional derivatives. This C-code can be compiled as a Dynamic-link Library (DLL) that can then be imported as an external function within the CasADi environment for use during the optimization.

```
/* This file was automatically generated by CasADi.
   The CasADi copyright holders make no ownership claim of its ...
       contents. */
#ifdef __cplusplus
extern "C" {
#endif

/* How to prefix internal symbols */
#ifdef CASADI_CODEGEN_PREFIX
  #define CASADI_NAMESPACE_CONCAT(NS, ID) ...
      _CASADI_NAMESPACE_CONCAT(NS, ID)
  #define _CASADI_NAMESPACE_CONCAT(NS, ID) NS ## ID
  #define CASADI_PREFIX(ID) ...
      CASADI_NAMESPACE_CONCAT(CODEGEN_PREFIX, ID)
#else
  #define CASADI_PREFIX(ID) foo_jac_ ## ID
#endif

#include <math.h>

#ifndef casadi_real
#define casadi_real double
#endif

```

```c
#ifndef casadi_int
#define casadi_int long long int
#endif

/* Add prefix to internal symbols */
#define casadi_f0 CASADI_PREFIX(f0)
#define casadi_f1 CASADI_PREFIX(f1)
#define casadi_f2 CASADI_PREFIX(f2)
#define casadi_s0 CASADI_PREFIX(s0)
#define casadi_s1 CASADI_PREFIX(s1)
#define casadi_s2 CASADI_PREFIX(s2)

/* Symbol visibility in DLLs */
#ifndef CASADI_SYMBOL_EXPORT
  #if defined(_WIN32) || defined(__WIN32__) || defined(__CYGWIN__)
    #if defined(STATIC_LINKED)
      #define CASADI_SYMBOL_EXPORT
    #else
      #define CASADI_SYMBOL_EXPORT __declspec(dllexport)
    #endif
  #elif defined(__GNUC__) && defined(GCC_HASCLASSVISIBILITY)
    #define CASADI_SYMBOL_EXPORT __attribute__ ((visibility ...
        ("default")))
  #else
    #define CASADI_SYMBOL_EXPORT
  #endif
#endif

static const casadi_int casadi_s0[6] = {2, 1, 0, 2, 0, 1};
static const casadi_int casadi_s1[5] = {1, 1, 0, 1, 0};
static const casadi_int casadi_s2[4] = {1, 1, 0, 0};

/* F:(i0[2])->(o0) */
static int casadi_f0(const casadi_real** arg, casadi_real** res, ...
    casadi_int* iw, casadi_real* w, void* mem) {
  casadi_real a0, a1, a2;
  a0=arg[0] ? arg[0][0] : 0;
  a1=cos(a0);
  a2=arg[0] ? arg[0][1] : 0;
  a0=(a0*a2);
  a1=(a1-a0);
  if (res[0]!=0) res[0][0]=a1;
  return 0;
}

CASADI_SYMBOL_EXPORT int F(const casadi_real** arg, ...
    casadi_real** res, casadi_int* iw, casadi_real* w, void* mem){
  return casadi_f0(arg, res, iw, w, mem);
}

CASADI_SYMBOL_EXPORT void F_incref(void) {
}

CASADI_SYMBOL_EXPORT void F_decref(void) {
}

CASADI_SYMBOL_EXPORT casadi_int F_n_in(void) { return 1;}
```

```
76
77  CASADI_SYMBOL_EXPORT casadi_int F_n_out(void) { return 1;}
78
79  CASADI_SYMBOL_EXPORT const char* F_name_in(casadi_int i){
80    switch (i) {
81      case 0: return "i0";
82      default: return 0;
83    }
84  }
85
86  CASADI_SYMBOL_EXPORT const char* F_name_out(casadi_int i){
87    switch (i) {
88      case 0: return "o0";
89      default: return 0;
90    }
91  }
92
93  CASADI_SYMBOL_EXPORT const casadi_int* F_sparsity_in(casadi_int ...
        i) {
94    switch (i) {
95      case 0: return casadi_s0;
96      default: return 0;
97    }
98  }
99
100 CASADI_SYMBOL_EXPORT const casadi_int* F_sparsity_out(casadi_int ...
        i) {
101   switch (i) {
102     case 0: return casadi_s1;
103     default: return 0;
104   }
105 }
106
107 CASADI_SYMBOL_EXPORT int F_work(casadi_int *sz_arg, casadi_int* ...
        sz_res, casadi_int *sz_iw, casadi_int *sz_w) {
108   if (sz_arg) *sz_arg = 1;
109   if (sz_res) *sz_res = 1;
110   if (sz_iw) *sz_iw = 0;
111   if (sz_w) *sz_w = 0;
112   return 0;
113 }
114
115 /* fwd1_F:(i0[2],out_o0[1x1,0nz],fwd_i0[2])->(fwd_o0) */
116 static int casadi_f1(const casadi_real** arg, casadi_real** res, ...
        casadi_int* iw, casadi_real* w, void* mem) {
117   casadi_real a0, a1, a2, a3;
118   a0=arg[0] ? arg[0][0] : 0;
119   a1=sin(a0);
120   a2=arg[2] ? arg[2][0] : 0;
121   a1=(a1*a2);
122   a3=arg[0] ? arg[0][1] : 0;
123   a3=(a3*a2);
124   a2=arg[2] ? arg[2][1] : 0;
125   a0=(a0*a2);
126   a3=(a3+a0);
127   a1=(a1+a3);
128   a1=(-a1);
```

```
129   if (res[0]!=0) res[0][0]=a1;
130   return 0;
131 }
132
133 CASADI_SYMBOL_EXPORT int fwd1_F(const casadi_real** arg, ...
        casadi_real** res, casadi_int* iw, casadi_real* w, void* mem){
134   return casadi_f1(arg, res, iw, w, mem);
135 }
136
137 CASADI_SYMBOL_EXPORT void fwd1_F_incref(void) {
138 }
139
140 CASADI_SYMBOL_EXPORT void fwd1_F_decref(void) {
141 }
142
143 CASADI_SYMBOL_EXPORT casadi_int fwd1_F_n_in(void) { return 3;}
144
145 CASADI_SYMBOL_EXPORT casadi_int fwd1_F_n_out(void) { return 1;}
146
147 CASADI_SYMBOL_EXPORT const char* fwd1_F_name_in(casadi_int i){
148   switch (i) {
149     case 0: return "i0";
150     case 1: return "out_o0";
151     case 2: return "fwd_i0";
152     default: return 0;
153   }
154 }
155
156 CASADI_SYMBOL_EXPORT const char* fwd1_F_name_out(casadi_int i){
157   switch (i) {
158     case 0: return "fwd_o0";
159     default: return 0;
160   }
161 }
162
163 CASADI_SYMBOL_EXPORT const casadi_int* ...
        fwd1_F_sparsity_in(casadi_int i) {
164   switch (i) {
165     case 0: return casadi_s0;
166     case 1: return casadi_s2;
167     case 2: return casadi_s0;
168     default: return 0;
169   }
170 }
171
172 CASADI_SYMBOL_EXPORT const casadi_int* ...
        fwd1_F_sparsity_out(casadi_int i) {
173   switch (i) {
174     case 0: return casadi_s1;
175     default: return 0;
176   }
177 }
178
179 CASADI_SYMBOL_EXPORT int fwd1_F_work(casadi_int *sz_arg, ...
        casadi_int* sz_res, casadi_int *sz_iw, casadi_int *sz_w) {
180   if (sz_arg) *sz_arg = 3;
181   if (sz_res) *sz_res = 1;
```

```
182   if (sz_iw) *sz_iw = 0;
183   if (sz_w) *sz_w = 0;
184   return 0;
185 }
186
187 /* adj1_F:(i0[2],out_o0[1x1,0nz],adj_o0)->(adj_i0[2]) */
188 static int casadi_f2(const casadi_real** arg, casadi_real** res, ...
        casadi_int* iw, casadi_real* w, void* mem) {
189   casadi_real a0, a1, a2, a3;
190   a0=arg[0] ? arg[0][1] : 0;
191   a1=arg[2] ? arg[2][0] : 0;
192   a0=(a0*a1);
193   a2=arg[0] ? arg[0][0] : 0;
194   a3=sin(a2);
195   a3=(a3*a1);
196   a0=(a0+a3);
197   a0=(-a0);
198   if (res[0]!=0) res[0][0]=a0;
199   a2=(a2*a1);
200   a2=(-a2);
201   if (res[0]!=0) res[0][1]=a2;
202   return 0;
203 }
204
205 CASADI_SYMBOL_EXPORT int adj1_F(const casadi_real** arg, ...
        casadi_real** res, casadi_int* iw, casadi_real* w, void* mem){
206   return casadi_f2(arg, res, iw, w, mem);
207 }
208
209 CASADI_SYMBOL_EXPORT void adj1_F_incref(void) {
210 }
211
212 CASADI_SYMBOL_EXPORT void adj1_F_decref(void) {
213 }
214
215 CASADI_SYMBOL_EXPORT casadi_int adj1_F_n_in(void) { return 3;}
216
217 CASADI_SYMBOL_EXPORT casadi_int adj1_F_n_out(void) { return 1;}
218
219 CASADI_SYMBOL_EXPORT const char* adj1_F_name_in(casadi_int i){
220   switch (i) {
221     case 0: return "i0";
222     case 1: return "out_o0";
223     case 2: return "adj_o0";
224     default: return 0;
225   }
226 }
227
228 CASADI_SYMBOL_EXPORT const char* adj1_F_name_out(casadi_int i){
229   switch (i) {
230     case 0: return "adj_i0";
231     default: return 0;
232   }
233 }
234
235 CASADI_SYMBOL_EXPORT const casadi_int* ...
        adj1_F_sparsity_in(casadi_int i) {
```

```
236   switch (i) {
237     case 0: return casadi_s0;
238     case 1: return casadi_s2;
239     case 2: return casadi_s1;
240     default: return 0;
241   }
242 }
243
244 CASADI_SYMBOL_EXPORT const casadi_int* ...
        adj1_F_sparsity_out(casadi_int i) {
245   switch (i) {
246     case 0: return casadi_s0;
247     default: return 0;
248   }
249 }
250
251 CASADI_SYMBOL_EXPORT int adj1_F_work(casadi_int *sz_arg, ...
        casadi_int* sz_res, casadi_int *sz_iw, casadi_int *sz_w) {
252   if (sz_arg) *sz_arg = 3;
253   if (sz_res) *sz_res = 1;
254   if (sz_iw) *sz_iw = 0;
255   if (sz_w) *sz_w = 0;
256   return 0;
257 }
258
259
260 #ifdef __cplusplus
261 } /* extern "C" */
262 #endif
```
